# The Effect of Heart Rate Variability Biofeedback Training on Vagal Tone in Athletically Talented Secondary School Students

**DOI:** 10.3390/sports10100146

**Published:** 2022-09-27

**Authors:** Andrea M. Firth, Torvald F. Ask, Stefan Sütterlin, Ricardo G. Lugo

**Affiliations:** 1 Department of Psychology, University Campus of Football Business-Wembley Stadium, Wembley, London HA9 0WS, UK; 2 Health, Welfare and Organization, Østfold University College, NO-1757 Halden, Norway; 3 Department of Information Security and Communication Technology, Norwegian University of Science and Technology, NO-2815 Gjøvik, Norway; 4 Faculty of Computer Science, Albstadt-Sigmaringen University, 72458 Albstadt, Germany

**Keywords:** talented athletes, vagally mediated heart rate variability, psychophysiological adaptability, focused breathing, heart rate variability biofeedback, vagal tone, autonomic nervous system, sports intervention

## Abstract

This study examines whether twelve sessions of heart rate variability biofeedback training would improve vagally mediated heart rate variability. If so, it would go some way in explaining why breathing-based interventions reduce clinical symptoms and improve non-clinical performance outcomes. Methods: Thirty participants (N = 30, N_female_ = 13) aged 14–13-year-old, all talented athletes, from a sport specialist school in SE London UK, were randomly divided into three groups, a control group, a psychology skills training combined with heart rate variability biofeedback training group, and a heart rate variability biofeedback only group. For the combined group, a variety of typical psychological skill training techniques were also used. Results: Paired participant t-test and the Wilcoxon Signed Rank test found non-significant differences between pre- and post-intervention measurements of heart rate variability. Non-significant results remained even after pooling the biofeedback training groups (*n* = 19). Conclusions: Our results do not indicate that beneficial effects associated with focused breathing training can be attributed to improved vagal tone. Further investigation into the underlying mechanisms of the benefits of focused breathing techniques is necessary to maximize clinical and non-clinical outcomes.

## 1. Introduction

**Figure 1:**
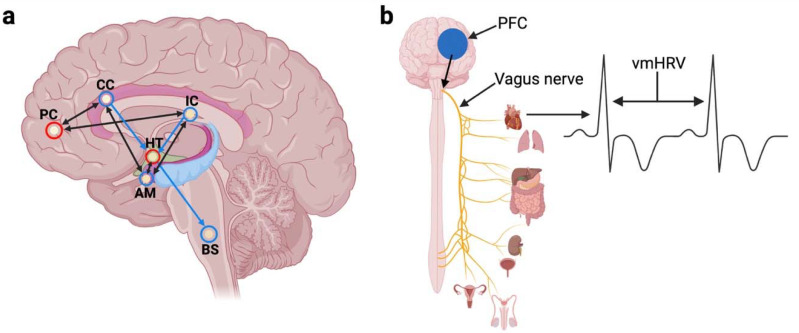
Prefrontally modulated vagal tone. (**a**) Simplified illustration of the neurovisceral integration model [9]. (**b**) Prefrontal input to the vagus nerve results in higher vmHRV. PC and PFC = Prefrontal cortex. CC = Cingulate cortex. IC = Insula. HT = Hypothalamus. AM = Amygdala. BS = Brainstem. vmHRV = Vagally mediated heart rate variability. Created with BioRender.com.

**Figure 2:**
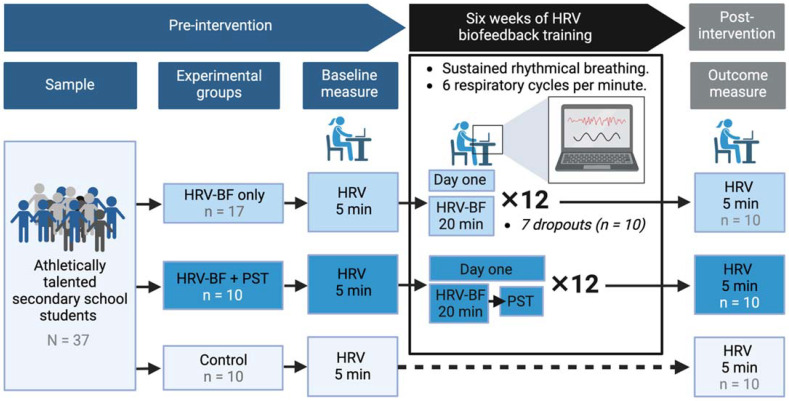
Overview of the experiment and training protocol. The original sample consisted of 37 participants. Seven of these dropped out during the HRV-BF training and were not included in the final analysis due to the repeated measures design. HRV-BF = Heart rate variability biofeedback. PST = Psychological skills training. Created with BioRender.com.

Vagally mediated heart rate variability (vmHRV), which is a quantification of the vagal influence on beat-to-beat variations in heart rate, has become an intensely investigated research topic in recent years. It is an increasingly accepted trait indicator of vagal tone and psychophysiological adaptability [1,2]. Low vagal tone has been associated with all-cause mortality [3,4,5] and health-related factors such as increased susceptibility to stress, increasing the risk of both cardiovascular and mental disease [6,7]. The model of neurovisceral integration (Figure 1a; [8,9]) describes vagal tone in a dynamical systems framework. Within this central-autonomic network (CAN; [10,11]), cognitive, emotional, behavioral, and physiological responses are modulated by pre-autonomic efferent activity initiated by prefrontal cortical areas. This is then mediated by subsequent activity in the sympathetic and vagal branches that innervate the heart [12]. The prefrontal modulation of sympathetic-parasympathetic interplay results in the cortico-cardiac interaction that is expressed as vmHRV (Figure 1b; [2]).

In recent years, vmHRV biofeedback (HRV-BF) has received increased attention in research [13] and commercial applications such as ALIVE (https://www.somaticvision.com (accessed on 10 August 2022).). Based on the evidence of associations between autonomic dysregulation and both physical and mental ill health [14,15], HRV-BF involves synchronization of breathing rate to the frequency at which HRV amplitude is maximized, and aims to increase vagal tone and consequently reduce clinical symptoms related to autonomic dysregulation. Most studies have applied the widely accepted treatment protocol by Lehrer and colleagues (see [16] for protocol description) and there are reports of considerable health benefits in terms of symptom reduction following HRV-BF [17].

Clinically significant health improvements using HRV-BF have repeatedly been reported at self-report and behavioral level for a range of physical conditions, including asthma [18,19,20,21], coronary heart disease [22,23,24,25], chronic obstructive pulmonary disease [26], fibromyalgia [27], chronic fatigue [28], and heart failure [29]. Improvements have also been reported for mental disorders such as major depressive disorder [6,30], post-traumatic stress disorder [31], subclinical anxiety [32] and stress-related chronic pain [33]. Although effect sizes are not regularly reported in the primary literature, moderate and large effect sizes were reported in two recent meta-analyses examining the efficacy of HRV-BF training in relieving symptoms of depression [30], stress, and anxiety [34], respectively. Positive results have also been reported in non-clinical settings such as improved performance in sports and scholastic performance following HRV-BF [35,36,37] and in sub-clinical settings where decreased blood pressure have been reported in pre-hypertensive individuals [38].

It has been suggested that the mechanism underlying symptom reduction after HRV-BF is a re-regulation of autonomic nervous system processes, with increased sensitivity of baroreceptors following repeated stimulation [18,39]. Supporting evidence for the HRV-BF induced neuroplasticity of the baroreflex comes from studies demonstrating baroreflex gain after HRV-BF in short term follow-ups [20,40]. These effects have been seemingly independent of whether participants have received just HRV-BF or the full protocol [20]. Baroreflex effects are typically calculated from the changes in heart rate corresponding to fluctuations in arterial blood pressure and represented as either baroreflex sensitivity or baroreflex effectiveness index (BEI; [41]). BEI quantifies the number of times the baroreflex successfully mediates changes in heart rate following blood pressure fluctuations. While it appears to be the best indicator of the baroreflex in humans [41], 24 h BEI monitoring suggests that only 21% of changes in heart rate following spontaneous fluctuations in arterial blood pressure is due to the baroreflex [42]. The remaining portions may be attributed to non-baroreflex mechanisms such as respiration, CAN activity, humoral substances, and chemoreflexes [41,42]. Although receptor-level functional plasticity in cardiac-autonomic neurons in the baroreflex arc has been observed in other pathological settings (e.g., [43]), direct experimental evidence on physiological processes responsible for baroreflex gain (e.g., altered receptor density) after HRV-BF training is currently lacking [44]. Only therapy-adjacent and total HRV-associated changes in brain functional connectivity has been reported after HRV-BF, but this was not associated with vmHRV indices [45].

Although the number of studies reporting improved clinical and psychological outcomes following HRV-BF training are growing, rather few studies report actual improvements in vagal tone. Short-term carry-over effects directly adjacent to an intervention of HRV-BF on total HRV have been reported (e.g., [24,45]. However, specific measures of vagal-cardiac activity did not show the expected increase post-treatment compared to pre-treatment. Intervention adjacent increases in vmHRV was reported in the HRV-BF intervention group of a controlled randomized pilot study following 10 sessions of HRV-BF training [38]. The study reported persistent improvements in clinical outcomes at a 3-month follow-up, but did not report if increases in vmHRV continued. Furthermore, impressive effect sizes in vmHRV was reported in another pilot study after only three sessions per week over two weeks of paced breathing with sessions lasting approximately 25 min [46]. The study did not have a clinical control group; thus, it remains unclear whether the observed changes in vmHRV were attributable to the intervention or other effects. Moreover, few studies demonstrate sustained biofeedback-related increases in resting vagal tone between sessions. Although some short-term effects on low frequency (LF) band power and global HRV have been reported in-between or immediately after sessions [20,45], the actual target of intervention (vagal tone as indicated by vmHRV) and the main indicator of HRV-BF remain seemingly unchanged. Taken together, the limited reports of changes in vagal tone post HRV-BF training implies that attributions of improved clinical outcomes to increased vagal tone are by and large unfounded.

Determining the underlying mechanisms of HRV-BF efficacy is essential to maximize clinical and performance outcomes. The apparent discrepancy between increasing reports of symptom reduction and actual vagal tone improvement following HRV-BF warrant further investigation. Thus, in the present study, we wanted to examine whether (H_1_) HRV-BF training would improve vagal tone, indicated by vmHRV. Based on previous studies [17], we hypothesize that 12 training sessions of HRV-BF over 6 weeks will improve vmHRV. Previous studies mainly report on either time domain or frequency domain indices of vmHRV (e.g., [45]), thus, we wanted to include indices from both domains to not miss out on any effects. This is the second part of a larger study, which sought to explore if HRV-BF training, either on its own or in combination with psychological interventions would improve academic performance, self-efficacy, and self-regulation. In the main study (reported elsewhere; [35]), HRV-BF did on its own and combined with psychological interventions, have a positive impact on academic performance and self-efficacy but not on self-regulation.

## 2. Materials and Methods

### 2.1. Participants and Background of the School

Thirty participants (*n* = 30, n_female_ = 13) from a sports specialist secondary school in southeast London were used in this study. All were year nine students aged 13 to 14, having chosen their options for stage four of their education in preparation for their General Certificate of Secondary Education (GCSE; equivalent to US 10th scholastic year), or equivalent examinations. The exclusion criteria meant that all participants were included on the schools gifted and talented programme (In the UK, the term talented is often used interchangeably with gifted and indeed at times the government’s definition does little to alleviate this confusion with music being included in both the gifted category and the talented category performing artistry [47]. The Department of Children Schools and Families [48], defines giftedness and talent in the school context as “Children and young people with one or more abilities developed to a level significantly ahead of their year group (or potential to develop those abilities)” p11). Further to this exclusion criteria, the students were identified as athletically talented. They engaged in high level extracurricular training or competition, at least six hours per week. Participants had no obvious health issues based on self and parental reports.

The school had a catchment area which attracts a significant number of students below the national socio-economic norm, with a higher than average number qualifying for free school meals. The school had a higher than national average proportion of ethnic minority groups, who spoke English as a second language, the school purported that seventy-three languages other than English were spoken as first languages by students of the school. Opinion varies on the importance of such factors in their impact on academic achievement in the UK. It was found that, for fifteen year olds in England, Northern Ireland and Wales students were adept at overcoming any disadvantage associated with lower socio-economic background [49]. This view is contrary to the statement by Wilshaw in the OFSTED report [50], in which he argues that the most able students from lower socio economic groups are those most likely to underachieve.

However, with respect to the participants used in this study, such information could not be obtained due to data protection. This, and considering the small number of participants these factors were not considered. At the time of the study the school was in special measures (OFSTED used a four-point grading scale in which grade one is outstanding, grade two, good, grade three requires improvement and grade four is inadequate. “How OFSTED Inspects Further Education Teaching and Training”. GOV.UK, www.gov.uk/guidance/being-inspected-as-a-further-education-and-skills-provider) (accessed on 10 August 2022). However, by the time of its second OFSTED inspection, conducted part way through this study, it was officially taken out of special measures having reached the necessary standards set by OFSTED scoring, (three) satisfactory overall.

The researcher first had to gain permission from the head of the gifted and talented programme, the associate head teacher and ultimately the executive headmaster of the school. The school also had an associate head teacher who had shared (co-headship) but not equal responsibility as head teacher of the school.

### 2.2. Ethics Statement

Ethical approval was gained via the University of Greenwich’s ethics committee (project number UREC/10/11.3.5.1). Permission from the secondary school to conduct the study was first obtained from the gatekeeper, in this instance, the head of the gifted and talented (G&T) programme, who was also the head of PE. Following permission from the school, permission was obtained from parents. The purpose of the study was explained, and it was made clear that participation was voluntary, as was the right of withdrawal, without explanation, up to the point of data analysis. Written parental consent, and written student assent, was collected. The present study complies with the Declaration of Helsinki, International Ethical Guidelines for Biomedical Research Involving Human Subjects and the International Guidelines for Ethical Review of Epidemiological Studies.

### 2.3. HRV Biofeedback Training Protocol

The specific aim of the present study was to see if HRV-BF training significantly impacted on the various post intervention frequency and time domain measures of HRV. If HRV could be altered by training this might go some way to explaining why breathing based interventions work.

This study consisted of three cohorts: A control group, a HRV-BF only group and a combined psychological skills training (PST) and HRV-BF group. Previous research by the main author, indicated that, for groups incorporating HRV-BF, significant results were found when compared to the control group with regard to academic improvement [35]. The best results were found when HRV and PST were combined, thus we included a combined PST and HRV-BF group to see if psychological variables could impact results. The combined HRV-BF and PST group consisted of six males and four females (*n* = 10). The control group consisted of six males and four females (*n* = 10). The HRV-BF only group consisted of five females (one data set was omitted as an outlier) and five males (*n* = 9). Both HRV-BF groups combined consisted of 11 males and eight females (*n* = 19). The data was collected in the mornings before the start of the school day. The study took part over one term of a sports specialist school in SE London. To avoid the half-term break, the intervention period was six weeks. The HRV-BF training consisted of 12 sessions of HRV-BF training lasting for 20 min each, and was conducted either alone or in combination with PST. Figure 2 shows an overview of the experiment.

The PST protocol has been described elsewhere [35]. In short, it included learning performance enhancing strategies through improved self-regulation including self-talk, focused attention, goal-identification, imagery and cognitive structuring of outcomes.

Participants were encouraged to maintain sustained rhythmical breathing, along with positive emotional states via positive thoughts. They were taught to breathe as close as possible to the rate of six respiratory cycles per minute. Participants achieved this at different rates over the course of the intervention, and not all did so, but all improved on smoothness of breath. The same training instructions and pre and post data collection were delivered to the participants at similar times of day, i.e., in the morning before the start of school, prior to exercise or training, with the exception of two athletes who were swimmers, one male and one female, and therefore trained early in the morning. However, their training was consistent throughout the time period of the study. All the participants included completed the protocol. There were seven drop outs (the original sample size pre HRV-BF was 37) and incomplete data collection but these were excluded from the analysis due to the repeated measures design (the final sample size was 30).

As heart rate fluctuations may occur depending on the position of the participant, all participants were seated during HRV-BF training. For HRV data collection and biofeedback training, the portable Clinical Version of the Somatic Vision HRV and biofeedback system was used. Heart rate and breathing rate were displayed as smoothed lines.

### 2.4. Heart Rate Variability Data Collection

Resting HRV was recorded for five minutes pre- and post-intervention. Data was collected during the morning before the start of the school day. All participants were seated during data collection. From one individual to another, there are considerable differences in HRV analysis, making it quite difficult to compare one person to another. Substantial individual differences may occur during the acquisition of HRV data (particularly the high frequency element) according to; the mental effort made by the participant [51], the type of athlete [52], or between athletes and non-athletes [53]. Therefore, to ensure standardization, participants were encouraged to maintain sustained rhythmical breathing by following an onscreen pacer, along with positive emotional states via positive thoughts, as previously described. Although engaging in various athletic pursuits, all participants were athletes, thus minimizing athlete versus non-athlete effects. As far as possible, the researcher tried to ensure that environmental conditions were replicated. However, due to the school environment extraneous variables could not always be controlled, for example noise levels, the mood of the participant and normal school distractions occurred.

### 2.5. Heart Rate Variability Data Reduction and Analysis

All HRV indices were quantified according to established methods [54]. After an initial 60 s period of the five-minute recording, the following four minutes of recorded data was analyzed. Even though four minutes is a short amount of data to analyze HRV, a few minutes of recording can provide sufficient results [53].

Pre- and post-intervention data was accrued from time domain analysis of HRV based on normal to normal RR intervals, that is, the measurement of time between two successive R-waves of QRS complexes. Using the HRV software analysis Kubios, raw data comprising the standard deviation RR (SDNN) was calculated. SDNN is the standard deviation of the normal beat to beat interval expressed in milliseconds (NN), and has been found to be a reliable means of test–retest HRV for short recordings using portable devices.

Frequency domain data was analyzed from the autoregressive (AR) spectrum. (AR spectrum analysis is advantageous in that it copes well with artifacts resulting in smoother and more readable data [55]). AR spectrum analysis estimates results by concentrating on the percentage power of the high frequency (HF) component of HRV. HF HRV is known to correspond with respiratory sinus arrhythmia and indicates parasympathetic nervous system effects on the heart. AR spectrum analysis also estimates the percentage of the power of LF/HF, indicating the ratio of both parasympathetic and sympathetic activity (the sympathovagal balance). Meaning, the higher the LF/HF percentage, the higher the sympathetic activity. Kubios, normalizes these ratios. So, it is possible to compare pre and post data for each participant, as well as, between group data using the percentage of power from the AR spectrum estimation results.

### 2.6. Heart Rate Variability Indices of Interest

The HRV indices that reflect autonomic modulation of heart rate are widely used indicators of vagal and sympathetic tone [54,56]. Previous studies assessing the effect of HRV-BF training on vmHRV either report only time domain or frequency domain indices of HRV but not both (e.g., [45]). Thus, for the present study, we extracted both time domain measures and frequency domain measures of vagal tone to avoid missing effects in any of the domains. Of time domain indices, we were mainly interested in SDNN, the square root of the mean of the sum of squares of differences between adjacent NN intervals (RMSSD), and the percentage of successive NN intervals exceeding 50 ms (pNN50). Whilst time domain analysis elicits useful information, certain time domain measures used to indicate vagal tone such as RMSSD are also influenced by sympathetic input [57]. To account for this possible confounder, we also included a frequency domain index of HRV, specifically the HF component (HF HRV).

### 2.7. Statistical Analysis

Variables were presented as mean (M) ± standard deviation (SD). As both the HF HRV and RMSSD data were skewed, they were transformed and normalized. Thereafter, the HF logarithm (HF Log) and the RMSSD log was also analyzed. The data from one female participant from the HRV-BF only group had to be discarded due to it being an outlier. All subsequent analyses were performed on the SDNN, pNN50, RMSSD and log transformed RMSSD and HF HRV indices.

HF HRV and RMSSD are usually highly correlated [58]. Due to the potential effects of short recordings (five minutes) on the quantification of HRV indices, Pearson correlations were performed on log transformed variables to check for this correlation as an indicator of HRV index quality.

Repeated measures (RM) ANOVA were computed for data that satisfied normal distributions and both within subject and between subject findings were reported using Tukey’s post hoc test. If data was not normally distributed, the Wilcoxon Signed Rank test was applied for statistical comparison. All ɑ values were set at 0.05.

The initial analysis compared results between the HRV-BF only (*n* = 9), the HRV-BF and PST (*n* = 10), and the control group (*n* = 10). A second analysis was performed by pooling the HRV-BF groups (*n* = 19) and comparing results against the control group to assess whether the total effects of having a HRV-BF intervention would lead to increased HRV.

A priori power analysis was not conducted. In this instance, the sample comprised of a specific population from which we could obtain a small number of individuals. We were also under a time constraint of having to complete the data collection within a working school environment and restricted to a time period in which we could fit in the required six weeks without interruption (i.e., half term breaks).

All data were analyzed using JASP version 0.15 [59].

## 3. Results

Pre-training RMSSD and pre-training HF HRV (r = 0.682, *p* < 0.001), and post-training RMSSD and post-training HF HRV (r = 0.729, *p* < 0.001) were highly correlated. Descriptives and statistical analysis from the RM ANOVA between the HRV-BF and PST, control, and HRV-BF only groups, can be found in Table 1.

As indicated in Table 1, there were no significant within or between group differences on any measures of HRV-BF outcomes.

To further explore if having a HRV-BF intervention could improve HRV, the HRV-BF only and HRV-BF and PST groups were combined into a single intervention group (*n* = 19). RM ANOVA were computed to compare HRV-BF intervention to the control group. Having a HRV-BF intervention did not improve HRV on any of the measures (see Table 2).

## 4. Discussion

In the present study, we wanted to examine whether HRV-BF training would improve vagal tone, indicated by increased vmHRV. Previous studies have found that HRV-BF training can improve several clinical variables that are related to vagal tone (e.g., [17,34]), but only a few studies have reported actual improvements in vmHRV after HRV-BF training [24,38,46]. These studies have considerable limitations such as only reporting intervention-adjacent results [24,38], or lacking a clinical control group [46]. Consequently, the question regarding the efficacy of HRV-BF training on vmHRV is not answered. To address this issue, we had participants go through 20 min of twelve sessions of HRV-BF training over a period of six weeks. In contrast to previous studies inferring vagal tone improvements from the effects of HRV-BF on clinical or performance outcomes, our main aim was only to assess the effect of HRV-BF training on indicators of vagal tone. As an improvement from earlier studies (e.g., [45]), we included both time domain and frequency domain indices of vmHRV. As we failed to observe any increases in vmHRV in either the individual or combined HRV-BF groups after the intervention, our results do not support that HRV-BF training improves vagal tone.

Our findings are in stark contrast to those of Siepmann and colleagues [46] who reported an impressive effect size for increases in vmHRV after only three sessions of HRV-BF training. When explaining their results, Siepmann and colleagues suggested that the HRV-BF training may have enhanced cardiac-vagal tone “by evoking focused concentration in combination with emotional self-control (p. 199)”. Siepmann and colleagues [46] further refer to neural circuits involved in the adaptive control of emotion and goal-directed behavior, such as the prefrontal cortex and anterior cingulate cortex. They consider a relevant contribution of higher cognitive processes such as emotion regulation strategies. In a similar vein, Nolan and colleagues [24] discuss possible pathways mediating biofeedback effects by evoking cognitive-emotional responses and refer to Thayer and Lane’s [9] cardiac autonomic network model originally described by Benarroch [10].

In a more recent study that had a sample size equivalent to the present one, HRV-BF training significantly improved intervention-adjacent global HRV but not indicators of vagal tone in the intervention group [45], although a preliminary report of the study with a lower sample size found significant increases in vmHRV [60]. Both the preliminary version and the full study did, however, not assess if the effects were significant between the intervention group and the control group [45,60]. Additionally, the full study only reported effects for time domain indices of HRV [45] which may be influenced by sympathetic input [57], potentially reducing the ability to observe increases in vagal tone. As we included both time domain and frequency domain indices of vmHRV in the analysis of both the individual and pooled HRV-BF training groups, our study goes further than previous studies in assessing the effect of HRV-BF training on vagal tone. The control group in the study by Schumann et al. [45] played a mobile game. Games are known to hijack the autonomic nervous system in order to sustain attention [61,62]. This could very well explain why no changes were observed in the control group after HRV-BF training, confounding the efficacy of their intervention, as well as explaining the observation of inter-group differences in total HRV-related brain activity [45].

The notion that the findings of Siepmann and colleagues [46] and Nolan and Colleagues [24] could be attributed to activity in neural circuits responsible for emotional responding are corroborated by a recent study showing that cognitive emotion regulation strategies can evoke situation-dependent increases in vmHRV in some individuals depending on baseline psychological variables [63]. Further, non-biofeedback breathing interventions increase intervention-adjacent (state) vmHRV leading to downstream physiological effects such as reduced expression of molecules known to exacerbate mental and somatic illness [14]. A few recent randomized controlled trials comparing the effect of mindfulness meditation and HRV-BF on stress reduction found no significant differences between the two interventions [64,65]. As the majority of changes in heart rate following fluctuations in blood pressure can be attributed to non-baroreflex factors such as respiration rate, chemoreflexes, humoral factors, and top-down CAN influences on the heart [41,42], and as the only assessment of HRV associated brain activity following HRV-BF has been intervention-adjacent [45], it is impossible to infer the main pathway responsible for intervention efficacy with respect to the few studies that report any changes in HRV [24,45,46]. That is regardless of HRV being intervention-adjacent, vagally influenced, or otherwise.

That outcome-relevant results can be decoupled from changes in vagal tone is supported by a meta-analysis of vagal tone in relation to global domains of both emotional and non-emotional regulatory cognitive control [66]. In their analysis, Zahn and colleagues [66] found only a weak average effect size for a limited number of domains, specifically emotion regulation, attention control, and executive inhibition. Higher study quality was generally associated with lower effect size. Moreover, when adjusting for missing studies the effect size reduced to zero. Consequently, the authors assumed that the true effect size was below r = 0.15 [66]. Thus, the relationship between HRV-BF and clinical improvements [17,34] or psychological improvement [37], reported in previous studies could very well be attributed to higher-order self-regulatory processes not directly related to vagal tone. This could explain the discrepancy in studies reporting clinical improvements without reporting change in vmHRV.

### Limitations

There are a few limitations associated with this study. Five minutes is a short time for recording of heart beats to use as quantification of HRV [54]. However, as our RMSSD and HF HRV indices were highly correlated at both pre- and post-training time points, it suggests that the indices were of good quality [58]. While we successfully eliminated non-athlete versus athlete effects on vmHRV, it is accepted that the type of athlete influences HRV results [52]. Thus, future research should include participants from similar athletic activity to better examine inter-group and especially within group differences. To avoid confounds related to dosage, the control group should also have been engaged by the researcher in an inert activity, especially as we did not find significant effects of HRV-BF on either time domain or frequency domain indices of vagal tone. It is, however, important that the activity is not of a sort that commands the autonomic nervous system such as video games. Whilst logistical issues involved within a school environment, and the time intensive nature of the study meant that it was not possible to obtain large participant numbers, the number of participants may have been too small to adequately assess any positive results that could have occurred. This may be the case even for the pooled HRV-BF group, although positive results on the SDNN index assessed in the present study have been reported elsewhere using a smaller but equivalent HRV-BF training group [45]. However, due to the modest sample size, the findings in the present study should be interpreted with caution. Given that the participants were talented athletes and frequently engaged in physical activity, the scope for vagal tone improvement was, perhaps, more reduced than for normal populations.

## 5. Conclusions

Although there is substantial evidence that HRV-BF training can have a beneficial effect in several clinical and non-clinical settings, research has yet to provide evidence supporting that these effects can be attributed to improvements in vagal tone. In contrast to previous studies examining the effect of HRV-BF on clinical or performance outcomes, the main aim of the present study was to assess whether HRV-BF training would improve vagal tone, indicated by increases in vmHRV. As we failed to observe any improvements, our study adds to the recent growing literature that HRV-BF training has little or no effects on vagal tone. Further efforts are necessary to investigate underlying mechanisms of the clinical benefits of HRV-BF to maximize clinical and performance outcomes.

## Figures and Tables

**Table 1:** Descriptive Statistics and Statistical Analyses for the Control, HRV-BF only, and HRV-BF and PST groups.

	SDNN			
	Pre	Post	F_w_	ω^2^
Control (*n* = 10)	74.3 ± 23.7	77.5 ± 12.9	1.48	0.012
HRV-BF (*n* = 9)	72.3 ± 21	111.9 ± 76.8		
HRV-BF and PST (*n* = 10)	66.5 ± 14.9	71.3 ± 34.3		
Sample Totals	71 ± 19.7	86.1 ± 49.3		
F_b_	1.93			
w2	0.022			
	RMSSD ^†^			
	Pre	Post	W	ES
Control (*n* = 10)	68.5 ± 23.3	65.5 ± 15.3	30	0.091
HRV-BF (*n* = 9)	72.3 ± 21	111.9 ± 76.8	10	0.164
HRV-BF and PST (*n* = 10)	61.4 ± 21.2	55.1 ± 25.7	33	0.200
Sample Totals	67.2 ± 21.6	76.3 ± 50.8		
F_b_				
w2				
	HF HRV log			
	Pre	Post	F_w_	ω^2^
Control (*n* = 10)	3 ± 55	3 ± 30	0.81	0.000
HRV-BF (*n* = 9)	2.9 ± 29	3.2 ± 50		
HRV-BF and PST (*n* = 10)	2.9 ± 42	2.9 ± 45		
Sample Totals	2.9 ± 43	3 ± 42		
F_b_	0.54			
w2	0.000			
	pNN50			
	Pre	Post	F_w_	ω^2^
Control (*n* = 10)	39.5 ± 13.1	41.2 ± 11.6	1.29	0.035
HRV-BF (*n* = 9)	33 ± 12.9	39.8 ± 16.5		
HRV-BF and PST (*n* = 10)	34.2 ± 13.7	28.6 ± 12.8		
Sample Totals	82.3 ± 11.7	82 ± 13.5		
F_b_	1.81			
w2	0.020			

F_b_ = between subjects. F_w_ = within subjects. ± = M ± SD. ES = Effect size. ω^2^ = Effect size. HRV-BF = Heart rate variability biofeedback. PST = Psychological skills training. SDNN = standard deviation RR. RMSSD = the square root of the mean of the sum of squares of differences between adjacent NN intervals. HF HRV = High frequency component HRV. pNN50 = the percentage of successive NN intervals exceeding 50 ms. † = RM ANOVA not performed for non-parametric data, therefore only Wilcoxon Signed Rank Test was performed.

**Table 2:** Descriptive Statistics and Statistical Analyses for the Control group and the pooled HRV-BF group.

	SDNN			
	Pre	Post	F_w_	ω^2^
Control (*n* = 10)	74.3 ± 23.7	77.5 ± 12.9	0.778	0.000
HRV-BF (*n* = 19)	69.3 ± 17.8	90.7 ± 60.4		
Sample Totals	71 ± 19.7	86.1 ± 49.3		
F_b_	0.145			
w2	0.000			
	RMSSD			
	Pre	Post	F_w_	ω^2^
Control (*n* = 10)	68.5 ± 23.3	65.5 ± 15.3	0.780	0.000
HRV-BF (*n* = 19)	66.6 ± 21	82.1 ± 61.7		
Sample Totals	67.2 ± 21.6	76.3 ± 50.8		
F_b_	0.420			
w2	0.000			
	HF HRV log			
	Pre	Post	F_w_	ω^2^
Control (*n* = 10)	3 ± 55	3 ± 30	0.444	0.511
HRV-BF (*n* = 19)	2.9 ± 36	3 ± 48		
Sample Totals	2.9 ± 43	3 ± 42		
F_b_	0.446			
w2	0.510			
	pNN50			
	Pre	Post	F_w_	ω^2^
Control (*n* = 10)	39.5 ± 13.1	41.2 ± 11.6	0.045	0.834
HRV-BF (*n* = 19)	33 ± 13.0	33.9 ± 15.4		
Sample Totals	82.3 ± 11.7	82 ± 13.5		
F_b_	2.49			
w2	0.122			

F_b_ = between subjects. F_w_ = within subjects. ± = M ± SD. ω^2^ = Effect size. HRV-BF = Heart rate variability biofeedback. SDNN = standard deviation RR. RMSSD = the square root of the mean of the sum of squares of differences between adjacent NN intervals. HF HRV = High frequency component HRV. pNN50 = the percentage of successive NN intervals exceeding 50 ms.

## Data Availability

Due to privacy and ethical concerns, based on the young age of the participants neither the data nor the source of the data can be made publicly available.
